# Editorial: Microbes and cultural heritage: from biodiversity to applications

**DOI:** 10.3389/fmicb.2026.1829794

**Published:** 2026-04-01

**Authors:** Virginia Helena Albarracín, Erasmo Gámez-Espinosa, Mai Bingjie, Sandra Gabriela Gomez de Saravia, Fadwa Jroundi

**Affiliations:** 1 Laboratorio de Microbiología Ultraestructural y Molecular, Centro Integral de Microscopía Electrónica (CIME), Facultad de Agronomía, Zootecnia y Veterinaria, Universidad Nacional de Tucumán-CONICET NOA SUR, Consejo Nacional de Investigaciones Científicas y Técnicas (CONICET), Tucumán, Argentina; 2 CIDEPINT Research and Development Center in Paint and Coating Technology, Faculty of Engineering, CICPBA-CONICET-UNLP, La Plata, Argentina; 3 Key Laboratory of Silicate Cultural Relics Conservation, Ministry of Education, School of Cultural Heritage and Information Management, Shanghai University, Shanghai, China; 4 Department of Microbiology, University of Granada, Granada, Spain

**Keywords:** biofilm formation, conservation strategies, cultural heritage, historical sites, innovative microbial management, microbial colonization, microbiome analysis in heritage, preservation of cultural heritage

Cultural heritage (CH) objects are not inert witnesses of the past; they are dynamic ecological niches shaped by continuous interactions between materials, environmental conditions, and living microorganisms. Manuscripts, wooden artifacts, stone monuments, wall paintings, leather objects, and archaeological remains provide substrates for microbial colonization. These microorganisms can drive aesthetic alteration, material weakening, salt-mediated damage, enzymatic degradation, and pigment formation, threatening historical integrity. At the same time, they provide insights into biodiversity, ecological adaptation, and innovative conservation strategies.

The Research Topic “*Microbes and cultural heritage: from biodiversity to applications*” brings together 11 contributions that collectively advance the field of heritage microbiology. Rather than focusing solely on descriptive surveys of microbial diversity, these studies integrate ecological perspectives with conservation science. By combining modern analytical approaches with material and environmental studies, the contributions demonstrate that understanding microbial biodiversity is essential not only for diagnosing biodeterioration processes but also for designing sustainable and informed preservation strategies. Taken together, the studies illustrate the ongoing transition of heritage microbiology from a descriptive discipline toward a more predictive and application-oriented field.

## Cultural heritage as a substrate-specific microbiome

A central theme emerging from this Research Topic is that each heritage material hosts a distinct, substrate-driven microbiome. Material composition, physicochemical properties, microclimate, and human contact shape microbial community assembly. As a result, cultural heritage objects function as highly specialized microbial ecosystems shaped by both environmental and anthropogenic factors.

Zhou et al. investigated the characteristic “peachy spots” on ancient Kaihua paper. Their results revealed that bacteria, rather than fungi, were strongly associated with stain formation. Acid-producing and pigment-forming genera such as *Methylobacterium* and *Rubrobacter* were enriched in affected areas, suggesting that metabolic activity linked to acidification and pigment synthesis may drive discoloration processes in historical paper. This work highlights the importance of linking microbial identity with functional potential in order to clarify deterioration mechanisms. Similarly, Alonso-Reyes et al. conducted the first comprehensive survey on microbial communities associated with historical artifacts from Argentina's Museum of Independence. The study revealed structured microbial biofilms dominated by Gram-positive taxa, with particularly high diversity detected on a nineteenth- century albumen photograph. The protein-rich composition of photographic material likely created favorable conditions for microbial growth. In addition, the presence of human- associated microorganisms on frequently handled objects underscored the influence of visitor interaction on museum microbiomes. Archaeological contexts further expand this perspective. Chen et al., analyzed microbial communities associated with soils from the Sanxingdui sacrificial pits. Distinct bacterial and fungal taxa were linked to processes such as organic matter degradation and potential bronze biocorrosion. Differences between surrounding soils and artifact-associated samples indicated that burial activities and material composition shape localized microbial assemblages. Together, these studies demonstrate that cultural heritage materials support highly specialized microbial communities whose composition reflects both environmental context and substrate chemistry.

## Keystone taxa and functional mechanisms of biodeterioration

Beyond identifying microbial diversity, several contributions address the functional mechanisms that drive biodeterioration. Understanding these mechanisms is essential for developing effective conservation strategies. Ma, Zhang et al. investigated epilithic biofilms colonizing the Leizhou Stone Dog monuments. Their analysis identified phototrophic bacteria and nitrifying microorganisms as keystone taxa of these biofilms. The study suggested that metabolic activity related to nitrogen cycling and phototrophic growth plays an important role in the deterioration of stone surfaces. These findings emphasize that the intensity of biodeterioration depends not only on microbial diversity but also on the metabolic functions performed by dominant taxa.

Wall paintings represent another vulnerable substrate. In a comparative study of the Maijishan and Mogao Grottoes, Ma, Chen et al. reported that bacterial communities differed markedly between sites. These differences were primarily driven by variations in substrate composition and microclimatic conditions. Humid environments favored proliferation of Actinobacteria, whereas arid conditions selected xerotolerant microorganisms. The absence of a shared core microbial community between the two sites highlights the strong influence of environmental filtering in shaping wall-painting microbiomes. Complementing this work, Nir et al. documented extensive microbial colonization on sgraffito wall art. Their observations revealed complex biofilm composed of bacteria, cyanobacteria, and fungi interacting with plaster and cement substrates. The heterogeneous distribution of microbial growth across the surface further illustrated how local environmental conditions influence colonization patterns. Together, these studies highlight the need for long-term monitoring and site-specific evaluation when assessing biodeterioration risks. Organic materials were also examined from a functional perspective. Wang et al. studied traditional shadow puppetry artifacts composed primarily of collagen-rich hide. The authors identified microorganisms capable of degrading collagen, a process that can lead to severe structural damage in leather-based heritage objects. Importantly, the study also explored antimicrobial compounds capable of inhibiting dominant degraders, illustrating how functional microbial analysis can inform targeted conservation strategies.

## Environmental drivers and treatment-induced microbial shifts

Environmental conditions and conservation treatments themselves can influence microbial dynamics on heritage surfaces. Several contributions in this Research Topic highlight the importance of considering these ecological responses. Tichy et al. monitored microbial changes associated with salt-removal treatments applied to historic buildings. Their results showed that shifts in salt composition altered the structure of microbial biofilms, with bacterial populations responding particularly strongly to these changes. The mineral composition also influenced both salt retention and microbial diversity. These findings demonstrate that conservation treatments may modify microbial ecosystems and therefore require careful ecological evaluation. In the earthen exhibition hall of the Terracotta Warriors Museum, Wang, Hou et al. examined how environmental factors shape fungal communities. Several genera, including *Penicillium, Aspergillus*, and *Talaromyces* were associated with specific microclimatic conditions. The authors also tested volatile allicin as a potential antifungal treatment, showing inhibitory effects on several strains. Such findings illustrate how environmentally compatible biocontrol strategies may help mitigate microbial growth on sensitive heritage materials.

## From diagnosis to innovation: microbial-informed conservation

Several studies in this Research Topic emphasize translational applications and the development of sustainable conservation strategies informed by microbiological knowledge. Kraśnicki et al. evaluated the effectiveness of ethanol mist treatment for disinfecting historical leather artifacts from the Auschwitz-Birkenau State Museum. The treatment significantly reduced microbial contamination while preserving the structural integrity of the leather. This study demonstrates that carefully designed disinfection strategies can successfully balance microbial control with material conservation. Dumbravă et al. explored protective coating for wooden objects incorporating silver nanoparticles within polymeric materials. These coatings showed antimicrobial and anti-biofilm properties while maintaining compatibility with the treated surfaces. Such innovations highlight the growing convergence between microbiology, materials science, and conservation technology.

Together, these applied studies signal an important shift in conservation practice. Rather than relying on broad sterilization approaches, modern strategies increasingly aim to manage microbial communities in ways that are compatible with both the material substrate and the surrounding environment.

## Toward a visionary and sustainable heritage microbiology

The contributions gathered in this Research Topic demonstrate that heritage microbiology has become an integrative discipline linking biodiversity research with conservation practice. Cultural heritage materials can be understood as dynamic microbial ecosystems shaped by environmental conditions, material properties, and human activity.

Climate change adds further urgency to this field of research. Intensified rainfall–drought cycles, salt crystallization events, and fluctuating humidity increasingly reshape microbial ecosystems on cultural heritage surfaces. Microbial communities respond rapidly to these stresses, often shifting toward halotolerant, stress-adapted, or pigment-producing taxa that can accelerate deterioration processes.

At the same time, microorganisms associated with heritage materials are not merely agents of decay. They can also provide valuable information about historical production techniques, storage conditions, and environmental exposures experienced by objects over time. In this sense, microbial communities may serve as biological archives that complement traditional historical and archaeological evidence.

Ultimately, the contributions presented in this Research Topic illustrate the evolvement of heritage microbiology from a discipline focused primarily on documenting biodeterioration toward one capable of predicting, monitoring, and managing microbial processes in cultural heritage environments. Through continued interdisciplinary collaboration among microbiologists, conservators, material scientists, and archaeologists, this emerging field will play an increasingly important role in safeguarding humanity's material legacy.

